# Green Synthesis and Characterization of Silver/Chitosan/Polyethylene Glycol Nanocomposites without any Reducing Agent

**DOI:** 10.3390/ijms12084872

**Published:** 2011-08-02

**Authors:** Mansor Bin Ahmad, Mei Yen Tay, Kamyar Shameli, Mohd Zobir Hussein, Jenn Jye Lim

**Affiliations:** Department of Chemistry, Faculty of Science, Universiti Putra Malaysia, 43400 UPM Serdang, Selangor, Malaysia; E-Mails: tmyen_87@hotmail.com (M.Y.T.); kamyarshameli@gmail.com (K.S.); mzobir@science.upm.edu.my (M.Z.H.); jennjye87@yahoo.com (J.J.L.)

**Keywords:** silver nanoparticles, nanocomposites, chitosan, polyethylene glycol, green chemistry

## Abstract

This paper presents the green synthesis of silver nanoparticles (Ag NPs) in aqueous medium. This method was performed by reducing AgNO_3_ in different stirring times of reaction at a moderate temperature using green agents, chitosan (Cts) and polyethylene glycol (PEG). In this work, silver nitrate (AgNO_3_) was used as the silver precursor while Cts and PEG were used as the solid support and polymeric stabilizer. The properties of Ag/Cts/PEG nanocomposites (NCs) were studied under different stirring times of reaction. The developed Ag/Cts/PEG NCs were then characterized by the ultraviolet-visible (UV-Vis) spectroscopy, X-ray diffraction (XRD), transmission electron microscopy (TEM), scanning electron microscopy (SEM) and Fourier transform infrared (FTIR) spectroscopy.

## 1. Introduction

Nanoscale materials are structures ranging from 1 to 100 nm, as defined in the chemistry context, which have contributed to the development of nanoscience and nanotechnology at an exponential rate in recent years [1]. Nanomaterials often have a significant degree of difference in physico-chemical and biological properties to their macroscale counterpart in spite of the similar chemical compositions they possess [2].

Silver nanoparticles (Ag NPs) have emerged as one of the most intensively studied areas in the field of nanotechnology due to their well-known effectiveness in biomedical [3], electronic [4], catalytic [5] and optical applications [6]. Many methods have been established in preparing metal nanoparticles, such as chemical reduction [7,8], electrochemical [9], irradiation [10,11] and thermal decomposition [12], as well as the green chemistry route [13]. Huang *et al.* [14] reported the synthesis of different metal-chitosan nanocomposites (NCs) in aqueous solution by the reduction of corresponding salts with NaBH_4_. The green synthesis is a concept that is introduced to define the method used in synthesis, which is favored over solvent medium. This is because it is environmentally friendly and contains a reducing agent that is benign to the environment. Besides, it also utilizes a non-toxic stabilizer in forming Ag NPs [15,16]. In addition, Wei *et al.* [17] also carried out research on Cts-based silver NCs by reducing silver nitrate (AgNO_3_) salts with non-toxic and biodegradable Cts. It appeared that the exclusion of NaBH_4_ in the synthesis made it “greener” as compared to the method reported by Huang *et al.* [14].

In the process of synthesizing nanoparticles, a stabilizer is used to control the formation and dispersion stability of metal nanoparticles. For this purpose, polymers have been widely used as a particle stabilizer to control the particle growth, stabilize the metal dispersions and limit the oxidation of the particle [18,19]. A number of researchers have reported on the synthesis of polymer-silver NCs with numerous polymers [20]. Notably, Cts is a natural cationic biopolymer obtained from deacetylation of natural chitin, which consists of polymeric β-(1,4)-2-amino-2-deoxy-d-glucose [21,22]. Due to its excellent biocompatibility, biodegradability, non-toxicity and bioactivity properties, Cts has gained much attention. The fact that it is a potential polysaccharide resource makes its use preferable [23].

Polyethylene Glycol (PEG) is a water-soluble polymer with a general formula H(OCH_2_CH_2_)_n_OH. It is widely used in the mechanical as well as pharmaceutical and cosmetic industries. PEG is also a good stabilizer for Ag NPs based on the conclusions made by several research studies as mentioned in this paper [24–26]. In one of these research works, Luo *et al.* reduced AgNO_3_ in the presence of PEG. The researchers suggested that stabilization can be obtained due to the free polymer chains in solution, where formation of aggregates is denied because of steric hindrance. From their observation, they also proposed that increasing the molecular weight of the polymer would help in forming stable Ag NPs [27].

The chemical reduction method is commonly used to prepare Ag NPs in industrial applications because of its great advantages in generating high yields and readiness to perform the method [28]. However, this method is one of the conventional methods that employs many chemical agents, and thus, making it hazardous to environment. Following this, the green synthesis method is much more suitable to be used in preparing Ag NPs as it is relatively more environmentally friendly. Hence, based on the principle of green synthesis [29], Cts and PEG were used as the stabilizer and solid support to prepare the silver nanoparticles in this work.

In this work, we proposed the green synthesis method by reducing AgNO_3_ with different stirring times at a moderate temperature for the preparation of silver nanoparticles. The influence of the stirring times on the optical properties, structures and morphologies of silver nanoparticles was characterized by the ultraviolet-visible (UV-Vis) spectroscopy, X-ray diffraction (XRD), transmission electron microscopy (TEM), scanning electron microscopy (SEM) and Fourier transform infrared spectroscopy (FTIR).

## 2. Results and Discussion

In this work, the reaction mechanism was proposed following the Equations (1–2) below:

(1) $$\text{Cts}/\text{PEG}+{\text{Ag}}^{+}\to {\left[\text{Ag}/\text{Cts}/\text{PEG}\right]}^{+}$$

(2) $${\left[\text{Ag}/\text{Cts}/\text{PEG}\right]}^{+}\overset{T=60{}^{\circ}C}{\underset{\text{Different stirring times}}{\to }}[\text{Ag}/\text{Cts}/\text{PEG}]↓$$

In this project, Cts solution was first mixed with PEG solution to form Cts/PEG solution. Cts/PEG then reacted with the Ag^+^ ion to form metallopolymer [Ag/Cts/PEG]^+^ (Equation 1). This metallopolymer was stirred at a moderate temperature of 60 °C according to varying stirring times to form Ag/Cts/PEG nanocomposites (Equation 2). In this process, Ag^+^ was successfully reduced to Ag^0^ to form Ag NPs.

### 2.1. Optical Properties

**Figure 1.**
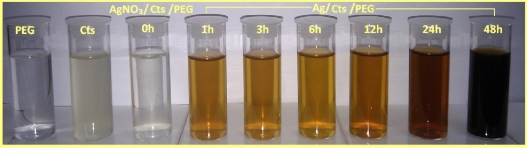
Photographs of polyethylene glycol (PEG), chitosan (Cts), silver nitrate (AgNO_3_)/Cts/PEG at 0 h and Ag/Cts/PEG nanocomposites (NCs) at different stirring times (1, 3, 6, 12, 24 and 48 h).

As shown in Figure 1, the color of the prepared AgNO_3_/Cts/PEG solution at different stirring times progressively changed from colorless to light brown, subsequently to brown, and eventually to dark brown. This phenomenon indicates the formation of Ag NPs in the Ag/Cts/PEG NCs solution [30].

**Figure 2.**
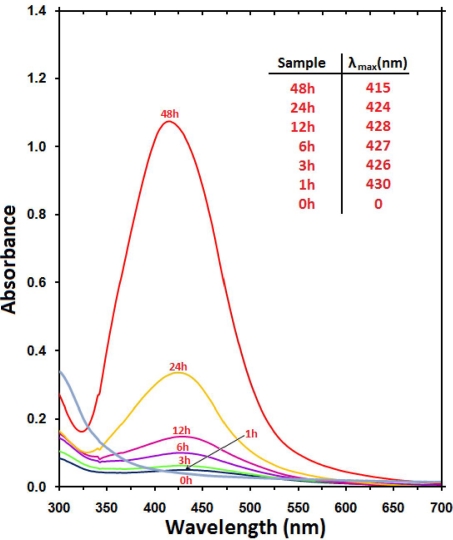
Ultraviolet-visible absorption spectra of AgNO_3_/Cts/PEG at 0 h and Ag/Cts/PEG NCs at different stirring times (1, 3, 6, 12, 24 and 48 h).

The formation of Ag NPs in the nanocomposites was further determined by using the UV-visible spectroscopy, which was shown on the surface plasmon resonance (SPR) bands. Figure 2 shows that Ag NPs started to form when AgNO_3_/Cts/PEG was allowed into reaction at a moderate temperature as there was no peak at 0 h and the absorbance peak could be seen at different stirring times after the reaction started. Generally, the SPR bands are influenced by the size, shape, morphology, composition and dielectric environment of the prepared nanoparticles [31,32]. Previous studies have shown that the spherical Ag NPs contribute to the absorption bands at around 400 nm in the UV-Vis spectra [33]. From this research, the SPR band characteristics of Ag NPs were detected around 415–430 nm (Figure 2), which strongly suggests that the Ag NPs were spherical. This can be confirmed by the TEM results. From Figure 2, as the stirring time of the nanocomposites increased, the intensity of the SPR peak also showed gradual increment. This shows that the reduction of the silver ions to silver atoms was continued and resulted in an increase in the concentration of Ag NPs [34]. However, there is an exceptional case in this situation for the SPR absorption band for the particles, which disagreed with the TEM results, whereby blue-shifts were observed as size decreased from d = 19.37 ± 4.98 nm and turned to red-shifts when nearing d = 5.50 ± 1.33 nm. This can be explained by the multi-layer Mie theory model, which theorizes that the chemical interaction caused the lowered electron conductivity in the outermost atomic layer, and consequently caused the red-shifts [35]. As seen from the figure, it can be observed that 48 h had very large absorbance compared to 24 h because the size of silver nanoparticles was a lot larger than those at 24 h. This phenomenon could be due to the fact that, after reaching a certain size, the stabilizer was not able to withhold the nanoparticle’s size effectively, which resulted in its very large size. Hence, the increase of size from 12 h (5.50 nm) to 24 h (6.45 nm) was very small as compared to the increase from 24 h (6.45 nm) to 48 h (19.37 nm) as demonstrated by the TEM.

### 2.2. X-ray Diffraction Characterization

**Figure 3.**
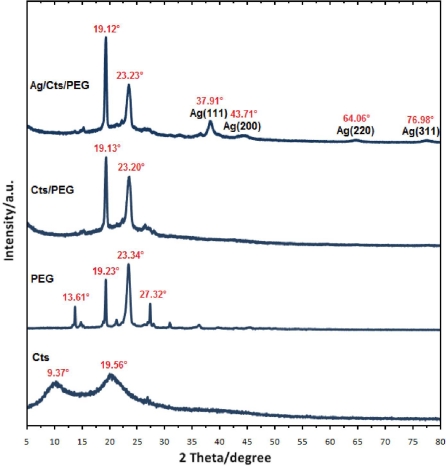
X-ray diffraction patterns of Cts, PEG, Cts/PEG and Ag/Cts/PEG NCs for the stirring time of 48 h.

The typical X-ray diffraction (XRD) patterns of the pure Cts, pure PEG, Cts/PEG and the prepared Ag NPs are shown in Figure 3. Pure Cts showed two peaks at 2θ of 9.37° and 19.56° while pure PEG showed strong reflections at 2θ of 19.23° and 23.34° and weak reflections at 13.61° and 27.32°. In Cts/PEG film, the 9.37° reflection for chitosan is diminished which may indicate that the crystallinity of chitosan is decreased. The diffraction of PEG tended to cover the reflection of chitosan with increasing reflection at 19.13° in Cts/PEG film. Therefore, it was observed that Cts/PEG showed strong reflections at 2θ of 19.13° and 23.20°. For Ag/Cts/PEG NCs, the XRD peaks at 2θ of 37.91°, 43.71°, 64.06° and 76.98° were characteristics to the (111), (200), (220), and (311) planes of the face-centered cubic (fcc) of Ag NPs, respectively [36]. The peaks showed that the main composition of nanoparticles was silver and no obvious other peaks present as impurities were found in the XRD patterns. Therefore, this gives clear evidence for the presence of Ag NPs in the Ag/Cts/PEG NCs.

### 2.3. Electron Microscopic Analysis

**Figure 4.**
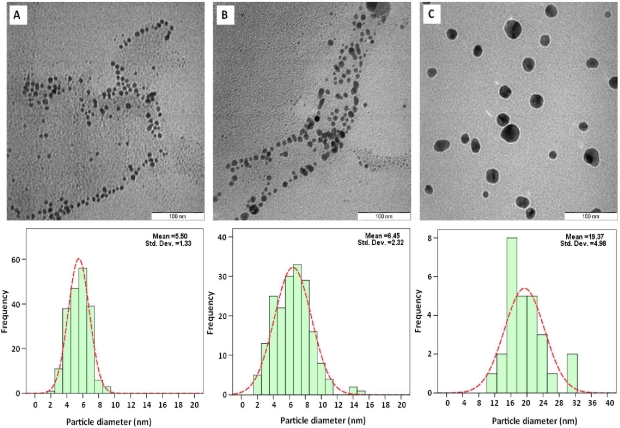
Transmission electron microscopy image and the particle size distribution for Ag/Cts/PEG NCs for the stirring times of 12, 24 and 48 h, respectively (**A**–**C**).

**Figure 5.**
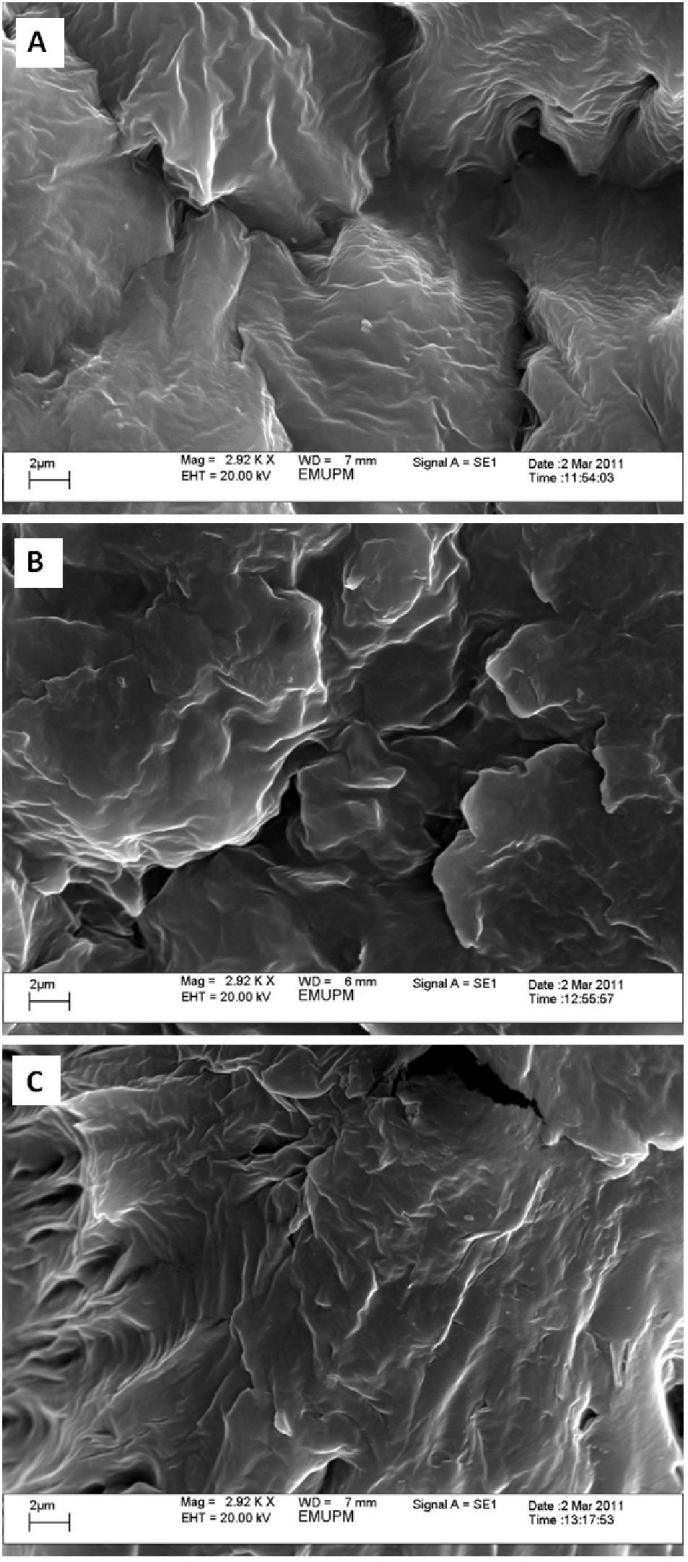
Scanning electron microscopy images of Ag/Cts/PEG NCs for the stirring times of 12, 24 and 48 h, respectively (**A**–**C**).

Figure 4 presents the transmission electron microscopy (TEM) images and the particle size distribution for Ag/Cts/PEG NCs with different stirring times of reaction. From the TEM results, the prepared sample with a longer stirring time had a broader particle size distribution. As seen from Figure 5, the Ag/Cts/PEG NCs with the stirring time of 48 h had a broad size distribution and a mean diameter of 19.37 ± 4.98 nm (Figure 4C). As the stirring time of reaction decreased, the mean diameter of Ag NPs decreased dramatically to 6.45 ± 2.32 nm and 5.50 ± 1.33 nm for 24 h and 12 h, respectively (Figure 4B and 4A). There was a large significant increase in the particle size as compared to the samples with the stirring times of 24 h and 12 h. This was due to the fact that, when the time of reaction increased, the particle aggregation was being promoted to form larger particles [37]. These results showed that the diameters of Ag NPs were influenced by the stirring time of reaction. The results also revealed that the stirring time of 12 h was the optimum in order to obtain the smallest particle size of Ag NPs.

The scanning electron microscopy (SEM) images of Ag/Cts/PEG NCs with the stirring times of 12, 24 and 48 h, respectively (A-C) are presented in Figure 5. The image reveals the surface structure of Ag/Cts/PEG NCs, which changed under different stirring times of reaction. Under a stirring time of 12 h, small-flake surfaces presented separately in the exterior morphology of Ag/Cts/PEG NCs. However, with the increased stirring time of reaction, larger flake surfaces presented in the Ag/Cts/PEG NCs. This phenomenon shows that, under longer stirring times of reaction, Ag/Cts/PEG NCs with better compatibility were produced.

### 2.4. Fourier Transform Infrared Characterization

**Figure 6.**
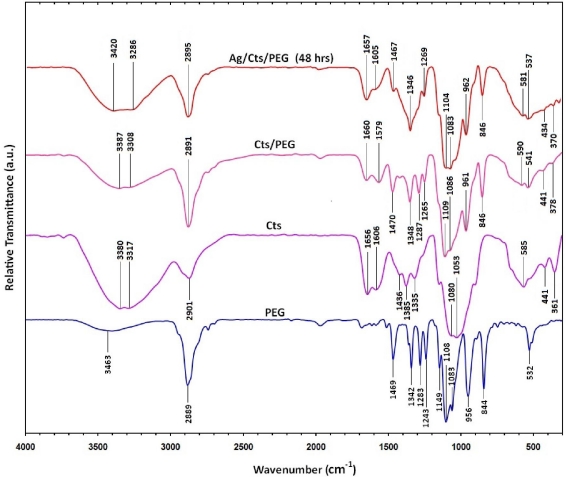
Fourier transform infrared spectra for PEG, Cts, Cts/PEG, and Ag/Cts/PEG NCs for the stirring time of 48 h.

Figure 6 shows the Fourier transform infrared spectra for PEG, Cts, Cts/PEG, and Ag/Cts/PEG NCs for the stirring time of 48 h. For the spectrum of PEG, the spectral band appeared at 3463 cm^−1^ for O-H stretching vibrations, 2889 cm^−1^ for C-H stretching vibrations, and 1469 and 1342 cm^−1^ for C-H bending vibrations. The absorption bands at 1283 to 1083 cm^−1^ were due to the stretching vibrations of the alcoholic O-H and C-O-C ether linkage. On the other hand, as for the Cts spectrum, the absorption bands at 3380 and 3317 cm^−1^ were due to the overlapping of O-H stretching and N-H stretching bands, 2901 cm^−1^ was due to the aliphatic C-H stretching, 1650 and 1606 cm^−1^ were due to N-H bending, 1436 and 1385 cm^−1^ were due to C-H bending, 1335 cm^−1^ was due to C-N stretching, and finally, 1080 and 1053 cm^−1^ were due to the overlapping of alcoholic C-O stretching band and ether linkage, as well as the C-O-C stretching band.

The Cts/PEG spectrum showed the combination of the IR absorption characteristic of PEG and Cts. The peaks of the overlapping O-H and N-H bands, aliphatic C-H and −NH_2_ groups in pure Cts were shifted to 3387, 3308, 2891, 1660, 1579 cm^−1^, respectively, in Cts/PEG. This was due to the deformation vibration of the amine group in Cts [38,39]. Few significant changes were observed in the Ag/Cts/PEG spectrum as compared to the Cts/PEG spectrum, except for the little shift in the spectrum and the disappearance of the peak at 1287 cm^−1^ for Cts/PEG. The disappearance of the small peak at 1287 cm^−1^ was replaced by the appearance of medium peak at 1346 cm^−1^ in the Ag/Cts/PEG spectrum, which was due to the complexation between Cts/PEG and AgNO_3_ to form metallopolymer [Ag/Cts/PEG]^+^.

**Figure 7.**
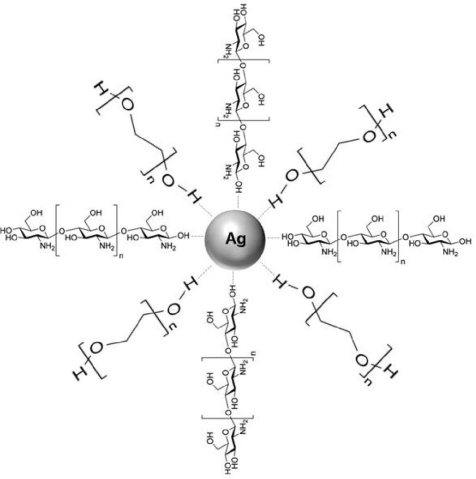
Schematic representation of Ag/Cts/PEG NCs synthesized from AgNO_3_/Cts/PEG by the green synthesis method.

The schematic representation of the Ag/Cts/PEG NCs synthesized from AgNO_3_/Cts/PEG by the green synthesis method is shown in Figure 7. The scheme represents the possible interactions between AgNPs and Cts/PEG based on the results predicted from the FTIR spectra.

## 3. Experimental Section

### 3.1. Materials

All chemicals and reagents were of analytical grade and used as received without further purification. AgNO_3_ was obtained from Fisher Scientific (Hong Kong). PEG (MW 1800–2200 g/mol) was purchased from Merck (Schuchardt, Germany). Low molecular weight Cts and Glacial Acetic acid (HAC, 99%) were obtained from Sigma Chemical, St. Louis, MO, USA. All the aqueous solutions were used with double-distilled water.

### 3.2. Synthesis of Ag/Cts/PEG NCs

For the synthesis of Ag/Cts/PEG NCs, the Cts solution was prepared by dissolved the Cts in 1.0 wt% of HAC solution and PEG-2000 solution was prepared by dissolved PEG-2000 in double-distilled water. The 50 mL of 1.0 wt% Cts solution and the 50 mL of 1.0 wt% PEG-2000 solution were mixed in the 100 mL conical flask. The 200 mg of silver nitrate (0.01 M) was added to this solution. The solution was then carried out under nitrogen atmosphere for 15 minutes to prevent oxidation reactions during formation of Ag NPs. The corresponding solution was stirred at 60 °C for stirring times of 1, 3, 6, 12, 24 and 48 h, respectively in a water bath to generate Ag NPs. The color of the solution started to convert from pale brown to dark brown, indicating the formation of Ag NPs. The reduction of AgNO_3_ into Ag NPs was monitored with UV-visible spectrophotometer. Finally, the obtained Ag/Cts/PEG NCs were made into thin films for further characterization.

### 3.3. Ag NPs Characterizations

The UV-visible spectral measurements were carried out using Shidmadzu UV-visible spectrophotometer (UV-1650PC-Tokyo, Japan) from 300 nm to 700 nm. The structures of the Ag/Cts/PEG NCs produced were studied using Philips X’pert Pro Panalytical PW3040MPD X-ray diffraction. Transmission electron microscopy observations were carried out using Hitachi H-7100 electron microscopy (Tokyo, Japan) and the particle size distributions were determined using UTHSCSA Image Tool version 3.00 programs. The surface morphologies of the Ag/Cts/PEG NCs produced were observed using LEO 1455 VPSEM scanning electron microscopy. The Fourier Transform infrared (FTIR) spectra were recorded with a Series 100 Perkin Elmer 1650 FTIR spectrophotometer (Walthman, MA, USA) over the range of 300–4000 cm^−1^.

## 4. Conclusions

Ag NPs were successfully synthesized by the green synthesis method with the use of green agents (Cts and PEG) under different stirring times of reaction at 60 °C. The formation of Ag NPs was confirmed in the UV-Vis absorption spectra, which showed the SPR band characteristics of Ag NPs in the range of 415–430 nm. The XRD result confirmed that the Ag NPs possessed a face-centered cubic crystal structure. In addition, this also revealed that Ag NPs were the main composition present in the nanocomposites without any contamination peaks. The structures and sizes of particles were characterized using TEM. The TEM images showed that the Ag NPs were in spherical shape and the average diameters of the particles were 5.50, 6.45 and 19.37 nm for the stirring times of 12, 24 and 48 h, respectively. In order to obtain the smallest particle size of Ag NPs, the results showed that a stirring time of 12 h was the optimum. On the other hand, the image of scanning electron microscopy revealed that, with the increased stirring time of reaction, larger flake surfaces presented in the Ag/Cts/PEG NCs. This also showed that Ag/Cts/PEG NCs with better compatibility were produced under longer stirring times of reaction.
